# Determinants of diarrheal diseases among patients attending public health centers in Addis Ababa and Hossana, Ethiopia: a matched case–control study

**DOI:** 10.1186/s41182-024-00675-4

**Published:** 2025-04-10

**Authors:** Deneke Wolde, Girmay Medhin, Haile Alemayehu, Genet Asfaw Tilahun, Kehabtimer Shiferaw Kotiso, Woinshet Hailu, Adane Mihret, Feyissa Regassa Senbato, Aklilu Feleke Haile, Tadesse Eguale

**Affiliations:** 1 Department of Medical Laboratory Science, College of Medicine and Health Sciences, Wachemo University, P.O.Box 667, Hossana, Ethiopia; 2 Aklilu Lemma Institute of Pathobiology, Addis Ababa University, P.O.Box 1176, Addis Ababa, Ethiopia; 3 Department of Midwifery, College of Medicine and Health Sciences, Wolkite University, Wolkite, Ethiopia; 4 Department of Public Health, College of Medicine and Health Sciences, Werabe University, Werabe, Ethiopia; 5 College of Health Sciences, Addis Ababa University, Addis Ababa, Ethiopia; 6 Armauer Hansen Research Institute, Addis Ababa, Ethiopia; 7 Department of Microbiology, Immunology and Parasitology, College of Health Sciences, Addis Ababa University, Addis Ababa, Ethiopia; 8 Ohio State Global One Health, Addis Ababa, Ethiopia

**Keywords:** Determinants, Diarrhea, Patients, Addis Ababa, Hossana, Ethiopia

## Abstract

**Background:**

The incidence of diarrheal diseases varies widely between and within countries due to different socioeconomic, environmental and behavioural factors. The aim of this study was to assess the determinants of diarrheal diseases among patients attending public health facilities in Addis Ababa and Hossana, Ethiopia.

**Methods:**

An age-matched case–control study was conducted in health facilities to recruit study participants and collect data from December 2021 to September 2022. Socio-demographic data and other risk factors were collected from study participants using a structured questionnaire. Conditional logistic regression was used to identify the independent predictor variables. The strength of the associations was measured using the adjusted odds ratio with the corresponding 95%CI. Statistical significance is indicated whenever the *p* value is less than 0.05.

**Results:**

Being partially vaccinated (AOR: 2.70; 95% CI 1.2, 5.9), use of tap water for drinking (AOR: 2.20; 95% CI 1.1, 4.4) and use of protected well/spring water for drinking (AOR: 13.90; 95% CI 3.7, 51.5), overcrowded sleeping places (AOR: 1.50; 95% CI 1.2, 1.8), contact with animal feces/food (AOR: 15.10; 95% CI 4.2, 53.6), the cleaning frequency of water-fetching materials (i.e., cleaned sometimes (AOR: 2.40; 95% CI 1.2, 4.5) and rarely (AOR: 3.03; 95% CI 1.2, 7.4)), and using an open latrine (AOR: 5.61; 95% CI 1.5, 21.0) were significantly associated with an increased likelihood of diarrhea. A higher BMI (AOR: 0.75; 95% CI 0.7, 0.8) was significantly associated with not having diarrhea.

**Conclusions:**

The incidence of diarrheal diseases was influenced by several factors, including children's immunization status and unhygienic living conditions. Therefore, timely immunization, access to safe drinking water, proper hygiene practices and improved sanitation facilities are essential for the control of diarrheal diseases and safeguarding public health.

## Introduction

Diarrheal diseases are a global health problem that affects people of all ages and both sexes [1]. In the general population, diarrhea is the third leading cause of morbidity and the sixth leading cause of mortality [2, 3] and accounts for 4.3% of the total global burden of disease [4]. Each year, approximately 1.6 million people worldwide die from diarrheal diseases, with the highest burden in developing countries [4]. In 2020, a total of 1,008 billion cases of diarrhea and 515,031 diarrhea-related deaths were reported in the African region [5]. In these regions, the death of one in eight under five children is due to diarrhea [6]. In Ethiopia, diarrheal diseases were among the leading causes of high disease burden at both national and regional levels. In 2019, 76.4 deaths from diarrheal diseases per 100,000 people were reported [7]. A systematic subnational analysis of the Global Burden of Disease Study found that in 2019, Addis Ababa had 105,243.37 cases and 24.42 deaths from diarrheal diseases per 100,000 population, while the Southern Nations, Nationalities and Peoples Region, which formerly included Hadiya Zone, had 126,151.96 cases and 83.72 deaths per 100,000 population [8].

The complex interplay of socioeconomic, environmental and behavioral variables determines the incidence of diarrheal diseases [9, 10]. Poor housing conditions such as overcrowding, dirty floors, inadequate food storage and living with pets are also associated with diarrheal diseases [11]. The majority of diarrheal diseases are due to unsafe drinking water supplies, inadequate sanitation and poor hygiene [12]. In developing counties, unsafe drinking water and inadequate sanitation and hygiene are leading causes of diarrhea [13]. More than 2 billion people worldwide do not have access to basic sanitation [14, 15], and Ethiopia has the worst access to safe drinking water and sanitation among sub-Saharan African countries [16, 17]. According to the 2019 Mini-Ethiopia Demographic and Health Surveys (EDHS), access to basic sanitation in Ethiopia is only 7.5% [18]. In 2023, access to basic sanitation and handwashing facilities in Hossana was reported at 35.1% and 16.8%, respectively [19]. The majority of the population in Addis Ababa lives in polluted environments and is thus exposed to water and sanitation-related diseases. A study conducted in Addis Ababa found that 53% of surface water sources and 62% of water source catchments are classified as very high-risk or high-risk contaminated [20].

The risk factors for this disease vary with age, the pathogens involved, the season in which the infection is acquired and the local environment [21]. Previous studies have been conducted in a single geographic area that may not reflect the broader population, and most previous studies in Ethiopia have focused on identifying risk factors in children under 5 years of age [22–24]. This study was conducted in two geographical locations with different socioeconomic and demographic characteristics in Ethiopia (i.e., Addis Ababa and Hossana) among all age groups to gain a comprehensive understanding of the determinants of diarrheal diseases among patients attending public health facilities.

## Methods

### Study setting and study period

This study was conducted in Addis Ababa city and Hossana town from December 2021 to September 2022. Addis Ababa is the capital city of Ethiopia, home to 23.8% of all urban residents in Ethiopia, and has an estimated population density of 5936.2 persons per square kilometre. There are 13 government hospitals and 98 health centers in the city.

**Fig. 1:**
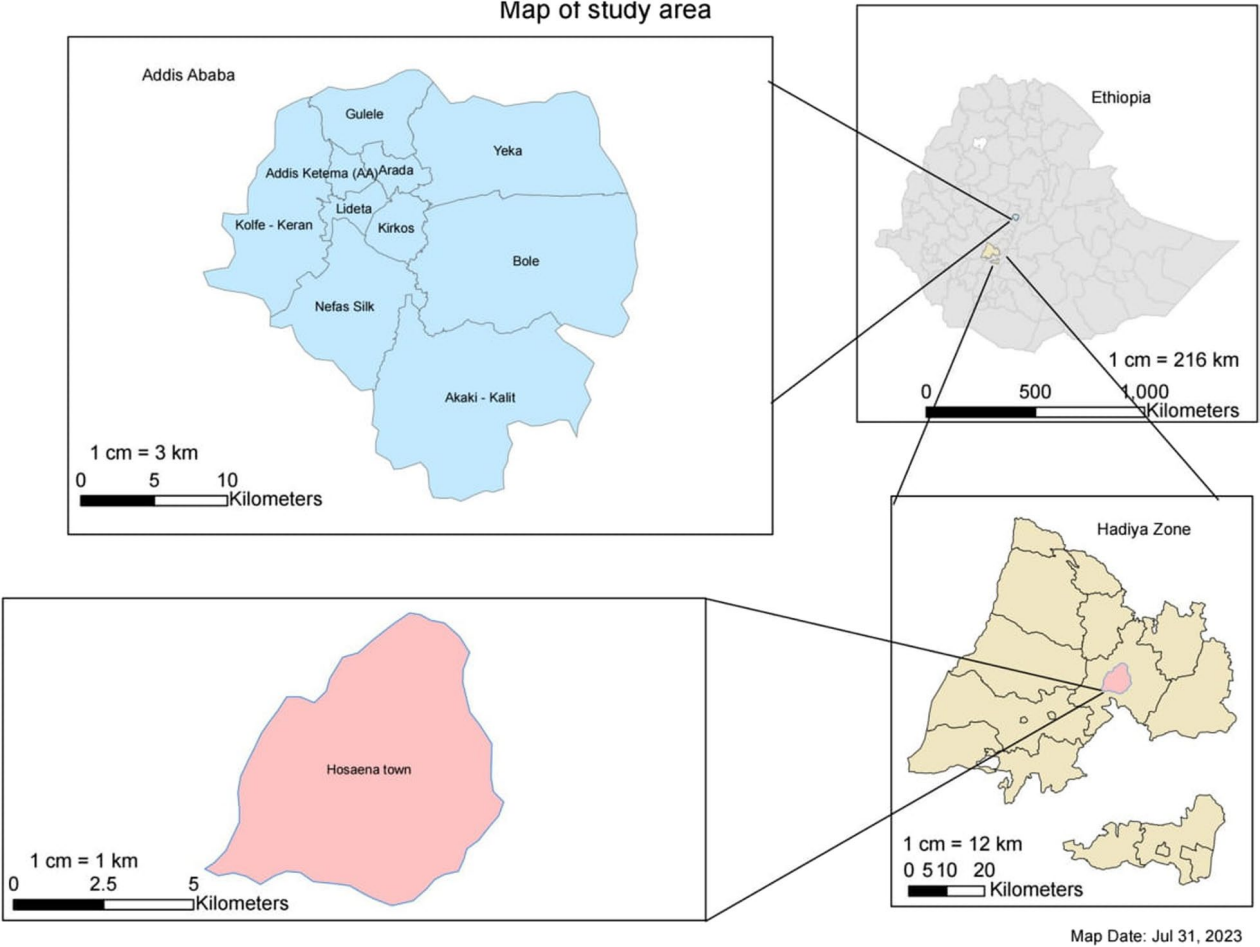
Map of Addis Ababa and Hossana town, Ethiopia

The other study area is the town of Hossana, located in the Hadiya zone in the Central Ethiopia region. The town is located 230 km from the capital Addis Ababa in the southwest. The zone is one of the most densely populated regions in Ethiopia. The population density of Hadiya zone is 415 persons per square kilometres. The total population of Hossana town is estimated at 75,963*.* The town has one government owned comprehensive specialized hospital, one private owned general hospital, three public health centers (primary healthcare facilities) and eight urban health extension workers offices. Figure 1 shows the map of the study area.

### Selection of health facilities for the current study

A multi-stage sampling method was used to select study participants in Addis Ababa. First, four sub-cities from Addis Ababa city administrative (which is 40% of the total) were selected. Then, 13 health centers (3 from Addis ketema, 4 from Arada, 4 from Kirkos, and 2 from Gulale sub-cities) were randomly selected based on the number of health centers available in the sub-cities. Finally, study participants from the health facilities were recruited proportionally according to patient flow in the randomly selected health facilities. In Hossana all three public health centers were included and participants were recruited proportionally according to patient flow like Addis Ababa.

### Study design and study participants

A health institution-based matched case–control study design with 1:1 ratio was employed. Case and control were matched by age (maximum of 3 years of age difference). Persons of any age who had diarrhea and attended health facilities selected for the current study were considered as cases. Patients with no symptom of diarrhea or gastrointestinal complaint for 2 weeks before enrolment in the study attending the same health facility were recruited as a control. Consecutive cases of diarrheic patients of any age and sex group who visited the health facilities during the data collection time were included. Comparison groups were selected from the same health facilities that do not have symptoms of diarrhea, who visited the facilities for other health purposes during the study period, and for each case was age match control. Controls were recruited within the same week after the recruitment of each diarrhea case. Any participants who had a sibling recruited as a case or control were excluded from the study. Potential controls who had diarrhea in the previous 7 days before the initiation of the study were also excluded.

### Sample size estimation and sampling procedure

The sample size was calculated using Stata version 14.0, considering a 95% confidence level, 90% power, a case-to-control ratio of 1:1, OR = 2.68, a design effect of 2 for Addis Ababa only and the proportion of controls with exposure = 35%. The odds ratio and proportion for controls were taken from the solid waste disposal category of a study conducted in Ethiopia by Mosisa et al. in 2021 [23]. After adding a 10% non-response rate, the final sample size for cases and controls in Addis Ababa was 488 (244 cases and 244 controls). For Hossana, we used 95% confidence level, 90% power, a case-to-control ratio of 1:1, an OR of 2.68, and a proportion of controls with exposure = 35%. However, we did not consider design effect assuming the clustering is minimal. The odds ratio and proportion for controls were again taken from the solid waste disposal category of the same study by Mosisa et al., resulting in a final sample size of 244 (122 cases and 122 controls). Overall, the total sample size used for assessing the determinants of diarrhea was 732 (366 cases and 366 controls).

### Sampling procedures of study participants

The total sample size for Addis Ababa was distributed to health facilities proportional to their patient flow. Consecutive cases of diarrheic patients of any age and sex group who visit the health facilities during the data collection time were included. Comparison groups were selected from the same health facilities that do not have symptoms of diarrhea, who visited the facilities for other health purposes during the study period, and for each case was age match control. Nurses who were work in a triage in each healthcare facility recruited the cases and controls for the study.

### Data collection methods and instruments

#### Demographic data

Data on socio-demographic, clinical data and other risk factors were collected through face-to-face interview using a structured questionnaire. The questionnaire was prepared in English, translated into Amharic, and finally, translated back to English to check its consistency.

#### Anthropometric measurements

Weight and height of study participants were measured at enrolment to determine the influence of anthropometry on the occurrence of diarrheal diseases. The standard procedure and measuring scale was utilized to determine the height. The height of infant age 6–23 months was measured in recumbent position, while height of the other study participants was measured in standing position. The study participants were requested to take off their shoes before taking height measurement. Height measurement was taken to the nearest of 0.1 cm. The calibrated beam balance was employed to determine the weight of the participants, and prior to each measurement, the scale was verified to ensure it was set to zero. Weight was measured to the nearest 10 g by removing heavy cloths. For a child who cannot stand alone, the mother was weighed together with a child and then without the child. The difference between the two measurements was taken as the child’s weight.

### Data quality and management

Quality of the data was assured using standardization of anthropometric measurements, providing adequate training for data collectors and supervision during the field work.

### Statistical analysis

Data analysis was done by Stata version 14.0. Continuous variables were described based on their distribution, either using the mean (standard deviation) or median (interquartile ranges), and categorical variables were reported as a number and percentage. The Shapiro–Wilk test was used to evaluate the normality of the quantitative variable data, and the variance inflation factor (VIF) test was used to check multi-collinearity. Conditional logistic regression was used to identify independent predictors after checking all the preliminary assumptions of the model. Variables with *p* value less than 0.25 in a bivariable analysis were identified as candidate for multivariable model. In multivariable analysis, variables with *p* value ≤ 0.05 were reported as being statistically significant and AOR with 95% CI was reported as measures of the strength of the associations.

### Operational definitions

**Diarrhea**: is defined as having three or more loose or watery stool in a 24 h period.

**Completely vaccinated**: children were those who received all childhood vaccines according to Ethiopia national immunization program.

**Partially vaccinated**: children who have received one or more immunizations according to the National Immunization Schedule, but have not completed all of them.

## Frequency of cleaning water fetching devices

**Frequently**: cleaning fetching materials four to six times per week.

**Sometimes**: cleaning fetching materials one to three times per week.

**Rarely**: not cleaning fetching materials at least once a week.

## Results

### Socio-demographic and socio-economic characteristics

A total of 732 participants (366 cases and 366 controls) were included in the current study. The median (IQR) age was 18.0 (7.0–30.0) years for cases and 20.0 (8.0–30.0) years for controls, and significant proportions were from the 0–14 year age group (i.e., 42.9% of the cases and 41.5% of the controls). The median (IQR) income of cases’ and controls’ household was 5000 (3500–7071) and 6000 (4000–8000) Ethiopian birr, respectively. The mean (± SD) number of rooms in the house of the cases and controls was 2.16 (± 1.1) and 2.40 (± 1.1), respectively. Regarding household family size, 66.4% of cases and 68.1% of controls had ≥ 4 members. On average, 4.14 (± 1.5) persons live in the house of the cases and 4.25 (± 1.5) persons live in the house of controls. On average 2.48 (sd = 1.1) persons in the house of cases and 2.44 (sd = 1.1) persons in the house of controls share a single sleeping room (Table 1).

**Table 1:** Socio-demographic characteristic of study participants from Addis Ababa city and Hossana town, Ethiopia

Characteristics	Total		Addis Ababa		Hossana	
	No. Cases (%)	No. Control (%)	No. Cases (%)	No. Control (%)	No. Cases (%)	No. Control (%)
Sex						
Male	193 (52.7)	196 (53.5)	131 (53.7)	139 (57.0)	62 (50.8)	57 (46.7)
Female	173 (47.3)	170 (46.5)	113 (46.3)	105 (43.0)	60 (49.2)	65 (53.3)
Marital status						
Never married	157 (42.9)	152 (41.5)				
Single	86 (23.5)	88 (24.0)	62 (47.0)	65 (47.1)	24 (31.6)	22 (29.3)
Married	115 (31.4)	124 (33.9)	63 (47.7)	72 (52.2)	51 (67.1)	52 (69.3)
Divorced	8 (2.19)	2 (0.55)	7 (5.3)	1 (0.7)	1 (1.3)	1 (1.3)
Educational status						
No formal education	30 (8.2)	22 (6.01)	22 (9.0)	10 (4.1)	8 (6.6)	12 (9.8)
Pre-school	46 (12.57)	39 (10.66)	34 (13.9)	28 (11.5)	12 (9.8)	11 (9.0)
Primary school (1–8)	121 (33.1)	142 (38.8)	73 (29.9)	88 (36.1)	48 (39.3)	54 (44.3)
Secondary school (9–12)	95 (25.96)	84 (22.95)	66 (27.0)	63 (25.8)	29 (23.8)	21 (17.2)
Above secondary school	74 (20.22)	79 (21.58)	49 (20.1)	55 (22.5)	25 (20.5)	24 (19.7)
Occupation						
Student	145 (39.6)	163 (44.5)	117 (48.0)	124 (50.8)	28 (23.0)	39 (32.0)
Government employee	55 (15.0)	67 (18.3)	35 (14.3)	47 (19.3)	20 (16.4)	20 (16.4)
NGO employee	5 (1.4)	4 (1.1)	2 (0.8)	1 (0.4)	3 (2.5)	3 (2.5)
Merchant	53 (14.5)	49 (13.4)	29 (11.9)	21 (8.6)	24 (19.7)	28 (23.0)
Other	108 (29.5)	83 (22.7)	61 (25.0)	51 (20.9)	47 (38.5)	32 (26.2)
Presence of under-5-year-old child in the household						
No under-five child	209 (57.1)	220 (60.1)	150 (61.5)	152 (62.3)	59 (48.4)	68 (55.7)
There are under five children	157 (42.9)	146 (39.9)	94 (38.5)	92 (37.7)	63 (51.6)	54 (44.3)
Immunization status						
Not vaccinated	2 (1.27)	1 (0.66)	2 (1.8)	0	0	1 (2.2)
Partially vaccinated	56 (35.67)	40 (26.32)	38 (35.2)	28 (26.4)	18 (39.1)	12 (27.3)
Fully vaccinated	99 (63.06)	111 (73.03)	70 (64.8)	78 (73.6)	28 (60.9)	32 (72.7)

### Environmental and behavioral characteristics

The presence of domestic animals in the households was reported by 26.2% of cases and by 18.4% of controls in Addis Ababa, and 38.5% of cases and 22.1% of controls in Hossana. Of the total animals reported, cats accounted for 70.3% of cases and 65.3% of controls. Similarly, dogs were reported by 42.3% of cases and 34.7% of controls.

In Addis Ababa, only 1.2% of cases and 0.4% of control reported using protected well or spring water for drinking. In Hossana, 23.0% of cases and 7.4% of controls reported using protected well or spring water as a source of drinking water. Overall, 18.0% of cases and 18.9% of controls treat their drinking water at home using various methods. More than half (56.5%) of cases and 36.9% of controls in Hossana used either an open pit latrine or a bush for defecation, while only 3.7% of cases and 1.6% of controls in Addis Ababa used these facilities for defecation. The average number of people sharing a toilet was 19.3 persons in the case group and 11.5 persons in the control group. The environmental and behavioral characteristics of the study participants are summarized in Table 2.

**Table 2:** Environmental and behavioral characteristic of study participants from Addis Ababa city and Hossana town, Ethiopia

Variables	Total		Addis Ababa		Hossana	
	No. Cases (%)	No. Control (%)	No. Cases (%)	No. Control (%)	No. Cases (%)	No. Control (%)
Animals living in the house with people	111 (30.3)	72 (19.7)	64 (26.2)	45 (18.4)	47 (38.5)	27 (22.1)
Where do animals stay at night?						
Inside dwelling	32 (28.8)	29 (40.3)	13 (20.3)	14 (31.1)	19 (40.4)	15 (55.6)
Outside dwelling	30 (27.0)	16 (22.2)	14 (21.9)	7 (15.6)	16 (34.0)	9 (33.3)
Both outside and inside	49 (44.1)	27 (37.5)	37 (57.8)	24 (53.3)	12 (25.5)	3 (11.1)
Children had close contact with animals	42 (65.6)	13 (31.0)	26 (63.4)	8 (29.6)	16 (69.6)	5 (33.3)
Child plays with animal feed, animal dung	45 (70.3)	10 (23.8)	25 (61.0)	7 (25.9)	20 (87.0)	3 (20.0)
Drinking water source						
Tap water	309 (84.4)	304 (83.1)	217 (88.9)	200 (82.0)	92 (75.4)	104 (85.2)
Protected well/spring	31 (8.5)	10 (2.7)	3 (1.2)	1 (0.4)	28 (23.0)	9 (7.4)
Bottled water	26 (7.1)	52 (14.2)	24 (9.8)	43 (17.6)	2 (1.6)	9 (7.4)
Households do something to make drinking water safer to drink	66 (18.0)	69 (18.9)	50 (20.5)	47 (19.3)	13 (10.7)	12 (9.8)
What do you usually do to the water to make it safer to drink?						
Boil	7 (10.6)	8 (11.6)	4 (7.7)	7 (13.0)	3 (21.4)	1 (6.7)
Add liquid chlorine	17 (25.8)	16 (23.2)	17 (32.7)	16 (29.6)	0	0
Use a water filter	41 (62.1)	44 (63.8)	30 (57.7)	31 (57.4)	11 (78.6)	13 (86.7)
Other	1 (1.5)	1 (1.5)	1 (1.9)	0	0	1 (6.7)
Household have rack for drying dishes in home	136 (37.2)	162 (44.3)	96 (39.3)	90 (36.9)	40 (32.8)	52 (42.6)
Toilet facility						
No facilities or use open pit latrine	78 (21.3)	49 (13.4)	9 (3.7)	4 (1.6)	69 (56.5)	45 (36.9)
Flush toilet	15 (4.1)	16 (4.4)	9 (3.7)	16 (6.6)	6 (4.9)	0
Ventilated improved pit (VIP) latrine	8 (2.2)	6 (1.6)	5 (2.0)	3 (1.2)	3 (2.5)	3 (2.5)
Pit latrine wit slab	265 (72.4)	295 (80.6)	221 (90.6)	221 (90.6)	44 (36.1)	74 (60.7)
How do you usually dispose of household rubbish?						
Rubbish pit	38 (10.4)	39 (10.7)	9 (3.7)	4 (1.6)	29 (23.8)	35 (28.7)
Put in garden	54 (14.8)	63 (17.2)	44 (18.0)	50 (20.5)	10 (8.2)	13 (10.7)
Put in bush	23 (6.3)	31 (8.5)	20 (8.2)	27 (11.1)	3 (2.5)	4 (3.3)
Open burning	91 (24.9)	99 (27.1)	37 (15.2)	39 (16.0)	54 (44.3)	60 (49.2)
Other	160 (43.7)	134 (36.6)	134 (54.9)	124 (50.8)	26 (21.3)	10 (8.2)
Patients who had known comorbidities	9 (2.5)	8 (2.2)	4 (1.6)	7 (2.9)	5 (4.1)	1 (0.8)
Patents who frequently wash hands	361 (98.6)	357 (97.5)	243 (99.6)	242 (99.2)	118 (96.7)	115 (94.3)
Patients who had habit of consuming raw vegetable	260 (71.0)	242 (66.1)	180 (73.8)	163 (66.8)	80 (65.6)	79 (64.8)
Patients who had habit of consuming raw milk	171 (46.7)	164 (44.8)	103 (42.2)	99 (40.6)	68 (55.7)	65 (53.3)
Household who had a habit of eating cooked food after its overnight storage	239 (65.3)	240 (65.6)	138 (56.6)	139 (57.0)	101 (82.8)	101 (82.8)

### Determinants of diarrheal disease among patients attending public health facilities

Multivariable binary logistic regression result showed use of tap water and use of protected well/spring water for drinking, child being partially vaccinated, a higher number of people sleeping in one room, contact with animal feces/feed, frequency of cleaning water fetching materials, increase in BMI level, and unavailability of toilet facility or use of open pit latrine as independent determinants of diarrhea (Table 3).

**Table 3:** Determinants of diarrheal disease among patients attending public health facilities in Addis Ababa city and Hossana town, Ethiopia

Characteristics	Number (%) without diarrhea	Number (%) with diarrhea	AOR (95% CI)	*p* value
Sex				
Male	196 (53.6)	193 (52.7)	1	
Female	170 (46.5)	173 (47.3)	1.38 (0.91, 2.10)	0.128
Income	6679.6(± 4614.7) #	6129.1(± 4582.5)#	1.00 (0.99, 1.00)	0.378
Children participants immunization status				
Fully vaccinated	111 (30.3)	99 (27.1)	1	
Partially vaccinated	40 (10.9)	56 (15.3)	2.70 (1.23, 5.91)	0.013*
Not vaccinated	1 (0.27)	2 (0.55)	11.53 (0.25, 320.3)	0.210
Education status				
No formal education	22 (6.0)	30 (8.2)	0.53 (0.20, 1.40)	0.202
Pre-school	39 (10.7)	46 (12.6)	0.48 (0.16, 1.42)	0.186
Primary (1–8)	142 (38.8)	121 (33.1)	0.35 (0.18, 0.69)	0.126
Secondary (9–12)	84 (23.0)	95 (26.0)	0.69 (0.39, 1.22)	0.198
Above secondary	79 (21.6)	74 (20.2)	1	
Number of living room	2.40 (± 1.14) #	2.16 (± 1.12) #	0.94 (0.77, 1.15)	0.523
Number of People sleeping in one room (mean ± SD)	2.8 (± 1.2)#	2.4 (± 0.9)#	1.55 (1.23, 1.92)	0.001*
Presence of animals living in a household				
No	294 (80.3)	255 (69.7)	1	
Yes	72 (19.7)	111 (30.3)	1.80 (0.95, 3.40)	0.070
Children Playing with animals, feeds or dung				
No	32 (76.2)	19 (29.7)	1	
Yes	10 (23.8)	45 (70.3)	15.1 (4.23, 53.6)	0.001*
Source of drinking water				
Tap water	304 (83.1)	309 (84.4)	2.20 (1.10, 4.40)	0.027*
Protected well/spring	10 (2.7)	31 (8.5)	13.90 (3.74, 51.50)	0.001*
Bottled water	52 (14.2)	26 (7.1)	1	
Type of toilet				
Flush toilet	16 (4.4)	15 (4.1)	1	
Ventilated improved pit (VIP) latrine	6 (1.6)	8 (2.2)	5.02 (0.74, 34.10)	0.100
Pit latrine wit slab	295 (80.6)	265 (72.4)	1.52 (0.50, 4.70)	0.467
Use open pit latrine	49 (13.4)	78 (21.3)	5.61 (1.50, 21.0)	0.010*
Having rack for drying dishes				
No	208 (56.8)	224 (61.2)	0.58 (0.34, 1.00)	0.051
Yes	158 (43.2)	142 (38.8)	1	
Frequency of cleaning water fetching material				
Daily	74 (20.2)	43 (11.8)	1	
Frequently	58 (15.9)	46 (12.6)	1.10 (0.45, 2.70)	0.834
Sometimes	205 (56.0)	247 (67.5)	2.35 (1.22, 4.51)	0.010*
Rarely	29 (7.9)	30 (8.2)	3.03 (1.24, 7.43)	0.015*
Contact with diarrheic patient				
No	362 (98.9)	352 (96.2)	1	
Yes	4 (1.1)	14 (3.8)	2.10 (0.56, 7.74)	0.276
BMI	18.6 (± 4.2)#	19.7 (± 3.5)#	0.75 (0.65, 0.83)	0.001*
Habit of eating raw vegetables				
No	124 (33.9)	106 (29.0)	1	
Yes	242 (66.1)	260 (71.0)	1.35 (0.88, 2.10)	0.170

^*^statistically significant (*p* < 0.05); # mean (+ SD)

## Discussion

This study was conducted to assess the determinants of diarrheal disease among patients attending public health facilities in Addis Ababa and Hossana. Preventable factors that include drinking water source, children's immunization status, overcrowded sleeping places, having contact with animal feces/food, cleaning of water fetching materials, unavailability of a toilet or use of an open pit, and BMI are significantly associated with likelihood of having diarrheal disease.

Although diarrheal diseases can be prevented by providing clean water, hundreds of millions of people in developing countries do not have access to clean water [25]. People in Ethiopia use both tap water and protected wells/springs as drinking water sources, which are significantly associated with diarrheal diseases in the current study. Patients using tap water or protected well or spring water for drinking were more likely to have diarrhea than patients using bottled water. This finding is consistent with a community-based comparative study among children under five in Jawi district, Awi Zone, Ethiopia [26], a study among children under five in Efoulan health district of Cameroon [27] and in a household in Mopani district of South Africa [28]. A study among children under the age of five in three districts in central Tanzania [29] showed a protective effect of drinking tap water against diarrhea. This may be related to the absence of a regular drinking water quality control system in Ethiopia, which highlights the risk of water contamination with a range of pathogenic microorganisms that cause diarrheal diseases. In addition, in most cases, water collection points are located some distance from the house, requiring water to be fetched and transported from the source and then stored in the household. As a result, unhygienic practices lead to water contamination during collection, transport, storage, and pickup. Moreover, the frequency of washing water-fetching materials was another important factor in determining the occurrence of diarrhea. This study found that patients who occasionally clean their water-fetching materials had a 2.40 times greater risk of developing diarrhea compared to those who clean them frequently. Similarly, the odds of developing diarrhea was 3.03 times higher among patients who rarely clean their water-fetching materials compared to those who do so regularly. This is in agreement with the study conducted in under-five children in Bosaso district, Puntland-Somalia [30]. This increased risk may be due to the fact that unclean fetching materials can become breeding grounds for harmful pathogens if they are not cleaned frequently. In addition, contaminants may interact with the surfaces of the containers, forming complex colonies allowing cells to survive in hostile environments, further increasing the risk of infection [31].

Sanitation is considered a primary barrier against infection as it excludes disease-causing microorganisms from the environment [32]. Unhygienic practices could contribute to over 23% of diarrheal illnesses [33]. In the current study, there is a significant association between the use of an open pit latrine and diarrheal diseases. It was found that patients who had no toilet facility or used an open pit latrine were 5.61 times more likely to have diarrhea than patients who used a flush toilet, which is consistent with other studies in Benishangul Gumuz Regional State in Ethiopia [34]; Dakahlia in Egypt [35]; Accra Metropolitan Area in Ghana [36]; and slum areas of Douala in Cameroon [37]. One possible explanation could be the likelihood of fecal contamination of the environment, which favors the spread of pathogens responsible for diarrhea. Moreover, poor sanitation practices allow human waste to enter drinking water supplies, contaminating nearby water sources and exposing the people to pathogenic microorganisms.

Adequate space is a fundamental element to support the maintenance of clean indoor air and reduce the risk of disease transmission. One of the factors associated with diarrheal disease in the current study is number of people sharing a bedroom. As the number of people sharing bedrooms increased by one, the odds of developing diarrhea increased by 55% which is in lines with the findings of previous studies in suburbs of Johannesburg, South Africa [38], in Salvador in northeastern Brazil [39], and in multisite study conducted in 8 countries [40]. The reason for this may be crowding creates an optimal environment for transmission of diarrheal pathogens and increases exposure to other risk factors associated with diarrhea.

Most pathogens causing diarrheal diseases in humans are zoonotic, and transmission of the pathogens can occur through contaminated food, water, the environment and direct contact with animals. In low- and middle-income countries, animals that harbor pathogens that can infect humans live in close proximity to them in domestic environments [41]. These conditions increase the potential for fecal contamination from animals in the home environment and subsequent zoonotic transmission of enteric pathogens carried by these food-producing animals. Animals in particular have been implicated as a source of fecal contamination of soil [42]. Closely related pathogens have been isolated from humans and animals, suggesting possible cross-infection between humans and domestic animals [43]. These pathogens are excreted in the feces of animals. Consequently, animal feces may contribute to the incidence of diarrheal disease in humans by introducing zoonotic pathogens that cause the disease or by increasing the transmission of pathogens found in both animals and humans [44]. Exposure of children to animal feces/feed was associated with an increased risk of diarrhea in this study. Compared to their counterparts, children exposed to animal feces/feed had a 15.1-fold higher risk of developing diarrheal disease. Similar to this finding, a systematic review by Penakalapati et al. [45] also showed that exposure to animal feces was associated with diarrheal illness. In addition, a consistent finding was obtained in a case–control study of children under 5 years of age in western Kenya matched for age, sex and place of residence [46]. This may be due to the fact that enteric pathogens are often shed in animal feces. Children's contact with animal feces can lead to fecal–oral transmission of these pathogens through fecal contamination of fingers [47].

The relationship between diarrhea and malnutrition is bidirectional. Malnutrition leads to an increased frequency and duration of diarrhea. This study found an association between BMI levels and diarrheal disease in patients attending public health facilities. Specifically, a 1 kg/m^2^ increase in BMI decreased the likelihood of developing diarrhea by 25%. This finding is consistent with previous studies in children under 5 years of age, which indicated that malnourished individuals were more likely to experience diarrhea in West Africa [48] and Namibia [49]. Malnutrition can predispose individuals to infection due to several physiological vulnerabilities and deficiencies in essential nutrients [50].

Vaccination is one of the most cost-effective means of public health interventions to prevent deaths from childhood infectious diseases, such as pneumonia and diarrheal disease. The full vaccination coverage among children in Ethiopia was 43% in 2019 [51]. This study identified childhood immunization status as one of the factors associated with diarrheal disease in patients attending public health facilities. The odds of partially vaccinated children compared to fully vaccinated children to have diarrhea is about three times. This finding is consistent with previous studies of children under 5 years of age in different regions of Ethiopia [52–54], in the Nyarugenge district of Rwanda [55], in the slums of Bankura in West Bengal [56] and in Epworth Township in Harare, Zimbabwe [57]. This may be due to the fact that vaccination significantly reduces morbidity and mortality from severe infections that disproportionately affect children [58]. Accordingly, vaccines developed against the most common causes of diarrheal disease contributed significantly to the decline in diarrheal disease [59]. Limitations of the study include that it relies on self-reported behaviour, which may not accurately reflect actual practices. In addition, the study only included patients who visit health care facilities, which may be systematically different from those who do not seek treatment. These limitations could restrict the generalizability of the results to the broader population.

In conclusion, unhygienic living conditions, vaccination status and BMI are potential predictors of diarrheal diseases. Therefore, efforts should be made to promote and ensure the timely vaccination of children and improve water safety and environmental and household hygiene in residential areas to reduce the incidence of diarrheal diseases and mitigate its impact.

## Data Availability

Data will be available from the corresponding author upon a reasonable request.

## Abbreviations

AOR: Adjusted odds ratio
BMI: Body mass index
IQR: Interquartile range
S: Standard deviation
WHO: World Health Organization
